# Genome-Wide QTL Mapping for Stripe Rust Resistance in Winter Wheat Pindong 34 Using a 90K SNP Array

**DOI:** 10.3389/fpls.2022.932762

**Published:** 2022-07-06

**Authors:** Xinli Zhou, Xin Li, Dejun Han, Suizhuang Yang, Zhensheng Kang, Runsheng Ren

**Affiliations:** ^1^School of Life Sciences and Engineering, Wheat Research Institute, Southwest University of Science and Technology, Mianyang, China; ^2^State Key Laboratory of Crop Stress Biology in Arid Areas, College of Plant Protection, Northwest A&F University, Xianyang, China; ^3^Excellence and Innovation Center, Jiangsu Academy of Agricultural Sciences, Nanjing, China

**Keywords:** wheat, stripe rust, QTL mapping, resistance gene, 90K wheat SNP array

## Abstract

Winter wheat cultivar Pindong 34 has both adult-plant resistance (APR) and all-stage resistance (ASR) to stripe rust, which is caused by *Puccinia striiformis* f. sp. *tritici* (*Pst*). To map the quantitative trait loci (QTL) for stripe rust resistance, an F_6−10_ recombinant inbred line (RIL) population from a cross of Mingxian 169 × Pingdong 34 was phenotyped for stripe rust response over multiple years in fields under natural infection conditions and with selected *Pst* races under controlled greenhouse conditions, and genotyping was performed with a 90K single nucleotide polymorphism (SNP) array chip. Inclusive composite interval mapping (ICIM) identified 12 APR resistance QTLs and 3 ASR resistance QTLs. Among the 12 APR resistance QTLs, *QYrpd.swust-1BL* (explaining 9.24–13.33% of the phenotypic variation), *QYrpd.swust-3AL.1* (11.41–14.80%), *QYrpd.swust*-*3AL.2* (11.55–16.10%), *QYrpd.swust-6BL* (9.39–12.78%), *QYrpd.swust-6DL* (9.52–16.36%), *QYrpd.swust-7AL* (9.09–17.0%), and *QYrpd.swust-7DL* (8.87–11.38%) were more abundant than in the five tested environments and *QYrpd.swust-1AS* (11.05–12.72%), *QYrpd.swust-1DL* (9.81–13.05%), *QYrpd.swust-2BL.1* (9.69–10.57%), *QYrpd.swust-2BL.2* (10.36–12.97%), and *QYrpd.swust-2BL.3* (9.54–13.15%) were significant in some of the tests. The three ASR resistance QTLs *QYrpd.swust-2AS* (9.69–13.58%), *QYrpd.swust-2BL.4* (9.49–12.07%), and *QYrpd.swust-7AS* (16.16%) were detected based on the reactions in the seedlings tested with the CYR34 *Pst* race. Among the 15 QTLs detected in Pindong 34, the ASR resistance gene *QYrpd.swust-7AS* mapped on the short arm of chromosome 7A was likely similar to the previously reported QTL *Yr61* in the region. The QTLs identified in the present study and their closely linked molecular markers could be useful for developing wheat cultivars with durable resistance to stripe rust.

## Introduction

Stripe rust, which is caused by *Puccinia striiformis* f. sp. *tritici*, is one of the most destructive fungal diseases of wheat in many cool and temperate regions of the world (Wellings, 2011), with China representing one of the most seriously affected areas (Wan et al., 2004, 2007). The pathogen of stripe rust is capable of long-distance dissemination and produces large-scale epidemics in a relatively short time, particularly under disease-favorable environmental conditions (Chen, 2020).

Compared to control measures, such as chemicals and cultivation methods, growing resistant cultivars is the most economical, environmentally friendly, and effective method for mitigating the disease (Chen, 2013). However, wheat breeding for stripe rust resistance has faced severe obstacles, such as the high frequency of variation in stripe rust races, which results in the continuous appearance of new physiological races, and many main wheat varieties have a single source of resistance, resulting in the frequent loss of resistance in agricultural production (Chen, 2013, 2020; Zhao et al., 2016). Therefore, exploring new effective disease resistance genes and identifying molecular markers closely linked to resistance genes are very important prerequisites for future breeding programs.

To date, more than 83 *Yr* genes for resistance to stripe rust have been identified and officially named, and dozens of temporarily named *Yr* genes or quantitative trait loci (QTL) have been reported (Maccaferri et al., 2015; McIntosh et al., 2017). Wheat stripe rust resistance genes can be divided into different types according to different classification criteria. One of the main classification methods divides the genes into two categories: all-stage resistance (also called seedling resistance) and adult-plant resistance (Chen, 2005, 2013). All-stage resistance can be detected at the seedling stage and is expressed throughout all the growth stages. This type of resistance is generally controlled by a single or several major genes, and at present, most of the resistance genes identified belong to all-stage resistance. This type of resistance is specialized to physiological races; that is, the variation and evolution of physiological races often lead to the loss of disease resistance in a short period of time, meaning that the persistence and stability of this type of resistance cannot be ensured (Chen, 2013). In contrast, adult-plant resistance shows a disease susceptibility response in the seedling stage, and as the plant grows older and the weather becomes warmer, the resistance usually shows a low infection type, low severity, and slow disease development rate in the adult stage. Adult resistance is usually not specific to pathogen races, thus reducing the selection pressure of wheat varieties on stripe pathogen races, and it corresponds to lasting and stable disease resistance. According to previous research results, adult resistance is generally controlled by several micro-effect genes (Chen, 2013) and could also be controlled by major effect genes, such as *Yr52* (Ren et al., 2012) and *Yr59* (Zhou et al., 2014). Due to these characteristics of adult resistance, many researchers worldwide have utilized resistance in combination with all-stage resistance as the main direction of adult-plant breeding wheat varieties exhibiting disease resistance.

Pindong 34 is a winter wheat cultivar bred in 1990 by the Institute of Crop Variety Resources, Chinese Academy of Agricultural Sciences, and it exhibits a high level of resistance to stripe rust and most other wheat diseases and insect pests. In 2014, Zhou et al. (2014) mapped the seedling resistance gene *Yr61* using a population of 208 F_2_ plants and 128 derived F_2:3_ lines in a cross between Mingxian 169 and Pindong 34. To effectively utilize the high level of resistance against stripe rust in Pindong 34, it is necessary to determine the mode of inheritance and identify the molecular markers linked to stripe rust resistance. The objectives of the present study were to characterize the resistance in Pindong 34, map and identify genes for all-stage resistance and adult-plant resistance, and develop molecular markers linked to the two types of resistance genes.

## Materials and Methods

### Plant Materials

Pindong 34 was selected from the cross [(Yan 7578/81/128)//176(15)9-26/Dongda 2]. Although it has the characteristics of low plant height, high 1,000-grain weight, and large grains, Pindong 34 had not been widely used in production. The most important characteristic of Pindong 34 is that it possessed resistance to various stripe rust races based on seedling tests and adult tests conducted by previous researchers under both controlled greenhouse and field conditions (unpublished data). Pindong 34 was crossed as a male parent with Mingxian 169 (MX169), which has a genotype susceptible to stripe rust. An F_6:10_ recombinant inbred line (RIL) population consisting of 119 lines was developed from a single F_1_ plant, and it was used for genetic analysis and molecular mapping.

### Greenhouse Tests

A total of 119 F_6:10_ RIL populations derived from a cross between Pindong 34 and Mingxian 169 were included in each test. Seedlings were grown for 15 days in a rust-free growth chamber at a temperature cycle of 20 °C during daytime and 16 °C at night, with a photoperiod of 16 h. At the two-leaf stage, seedlings were uniformly inoculated with fresh urediniospores of race CYR34 with talc at a ratio of 1:20. Inoculated plants were kept in a dark dew chamber at 10 °C and over 100% relative humidity for 24 h for urediniospores to germinate and infect the plants, which were then moved to a growth chamber set to a diurnal cycle, with a gradual change in the temperature from 12°C to 16°C and a 16-h photoperiod, to enhance stripe rust development. The infection type was recorded 15 days after inoculation using the 0–9 scale (Line and Qayoum, 1992). Seedlings of MX169 were included as the susceptibility check.

### Field Tests

The 119 RIL families and parents were grown in natural environments of Mianyang (31°33′ N, 104°55′ E) of Sichuan province and Yangling (34°17′ N, 108°04′ E) of Shaanxi province in China from 2016 to 2020. The RILs were planted in a randomized block design with two replicates. The plots were 1 m long and 25 cm apart. Twenty seeds were planted in each row. Every 20 lines, the highly susceptible stripe rust wheat variety Mingxian 169 was planted, and in each environment around the plot, Mingxian 169 was also planted as an inducer. The soil conditions in the experimental fields were favorable for crop growth and development and grain filling.

Mianyang is located in southwestern China at an average altitude of 485 m above sea level, and is characterized by a mean annual rainfall of 823.3 mm (36 years average) and a mean annual temperature of 16.0 °C (36 years average). Mianyang is a natural over-wintering and over-summering region for stripe rust in China and nurseries regularly become infected without artificial inoculation. Yangling is located in northwestern China at an average altitude of 519 m above sea level, and is characterized by a mean annual rainfall of 635.1 mm per year and a mean annual temperature of 12.9 °C. Trials at Yangling were inoculated with a mixture of *Pst* races CYR32 and CYR34 suspended in liquid paraffin (1:300) and sprayed onto Mingxian 169 and Avocet S at flag leaf emergence. When the susceptible variety reached 90%−100%, the response type severity was recorded for both the Mianyang and Yangling district populations and their parents according to criteria from grades 0–9 as described by Line and Qayoum (1992).

### DNA Extraction

The DNA from the parents (Pindong 34 and Mingxian 169) and 119 RIL populations was isolated from young leaves after freezing in liquid nitrogen and grinding using a modified cetyltrimethylammonium bromide (CTAB) method (Li et al., 2013). After removing RNA with RNase and centrifugation, DNA was dissolved in Tris-EDTA buffer (10 mM Tris-HCl and 1 mM EDTA, pH 8.0). The DNA quality and concentrations were determined by electrophoresis and spectrophotometry (NanoDrop ND-1000, Thermo Scientific, Wilmington, DE, USA). The stock DNA solutions were diluted with sterilized ddH_2_O to different concentrations depending on the requirements of the individual experiment for further molecular analysis.

### STS Marker Analysis

Two STS markers were developed from the RGAP markers flanking the resistance gene *Yr61* reported in 2014 and screened for polymorphisms between the parents of the RIL population. The polymorphic markers were used to genotype the RILs. The distributions of the markers on wheat chromosome 7AS were determined based on Zhou et al. (2014). The STS markers *STS5467* (forward primer: 5′-GTAGGTTCTCGACTCCACACAC-3′ and reverse primers: 5′-GTTGTAGGAGCAGCGGAAGAAC-3′) and *STS5765b* (forward primer: 5′-ATCATCTCATTAGCGTTTAG-3′ and reverse primer: 5′-CTCTGCCTGTGCTTTCGG-3′) were used to detect the products of polymerase chain reaction (PCR) through direct labeling. The PCR mixture, amplification conditions, and product detection for STS markers included initial denaturation at 94°C for 4 min and 35 cycles of 94°C for 35 s, 55°C for 35 s, and 72°C for 35 s (Zhou et al., 2014).

### Genetic Map Construction and QTL Mapping

Genotype data from the DNA of the 119 populations was scanned by the 90K chip assay to perform the preliminary genotyping of missing and unexamined data. Marker deletion was performed using the “Bin” function in ICI Mapping v4.1 software, and genetic mapping was performed using ICI Mapping v4.1 software with the Kosambi mapping function (Kosambi, 2016).

The infection type (IT) and disease severity (DS) data of both the seedling stage and adult-plant stage in each year and location were used for QTL analysis and mapping, and QTL mapping was performed with ICI Mapping v4.1 software to detect possible QTLs. When the LOD value was >2.5, a QTL was identified, and the contribution rate and additive effect value of each QTL were calculated.

### Statistical Analyses

The phenotypic data were analyzed based on a variance analysis (ANOVA) in ICI Mapping v4.1 software for variance analysis and generalized heritability calculation. Broad-sense heritability (h^2^) of stripe rust resistance was estimated as ${\text{h}}^{2}={\text{σ}}_{\text{g}}^{2}$/(${\text{σ}}_{\text{g}}^{2}$ + ${\text{σ}}_{\text{ge}}^{2}$/r + ${\text{σ}}_{\text{ε}}^{2}$/re) (Wang, 2009; Meng et al., 2015), where ${\text{σ}}_{\text{g}}^{2}=$genotypic (line), ${\text{σ}}_{\text{ge}}^{2}=$genotype × environment interaction, ${\text{σ}}_{\text{ε}}^{2}=$ residual error variances, e=number of environments, and r=replicates per environment.

To determine the additive effects and combinations of QTLs, boxplots were plotted for the mean IT and mean DS values of the 119 RILs sharing the same number of beneficial alleles.

### Information Data for QTL Located Within the 10 Chromosome Physical Intervals

The locations of the QTLs identified in this study were compared with those of previously published resistance genes or QTLs to stripe rust to obtain the physical positions, reference sequences, and marker alignments of polymorphic SNP markers, and the SNP probes were aligned with the Chinese Spring sequence through a BLAST search (IWGSC Ref Seq v1.0; https://plants.ensembl.org/Triticum_aestivum/Info/Index).

## Results

### Phenotypic Characterization of ASR Resistance

**Figure 1:**
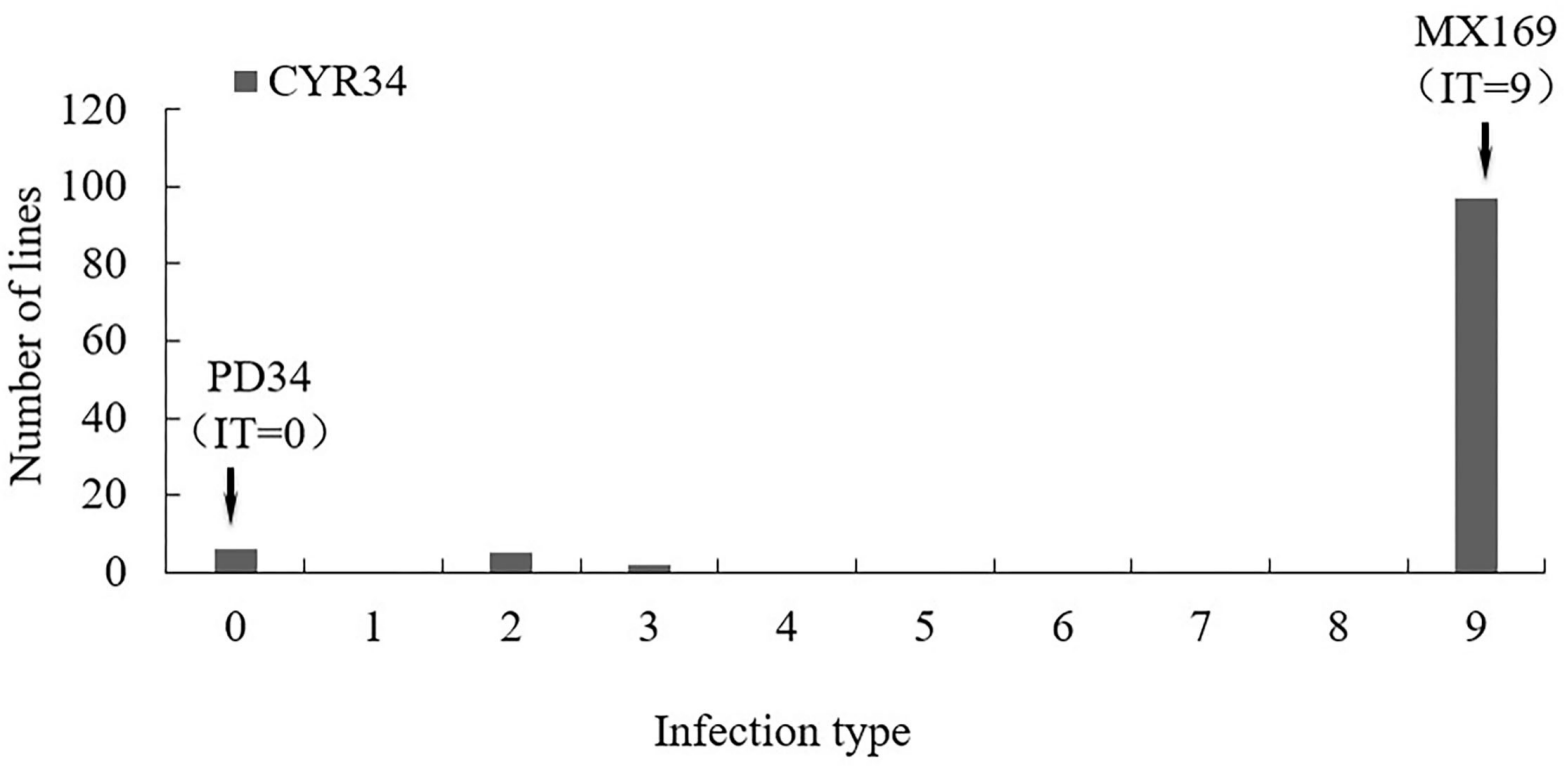
Frequency distributions of seedling infection type (IT) value in the Mingxian 169 (MX) × Pindong 34 (PD) derived recombinant inbred line (RIL) population tested with races CYR34 in greenhouse. Arrows indicate the values of the parental lines.

**Figure 2:**
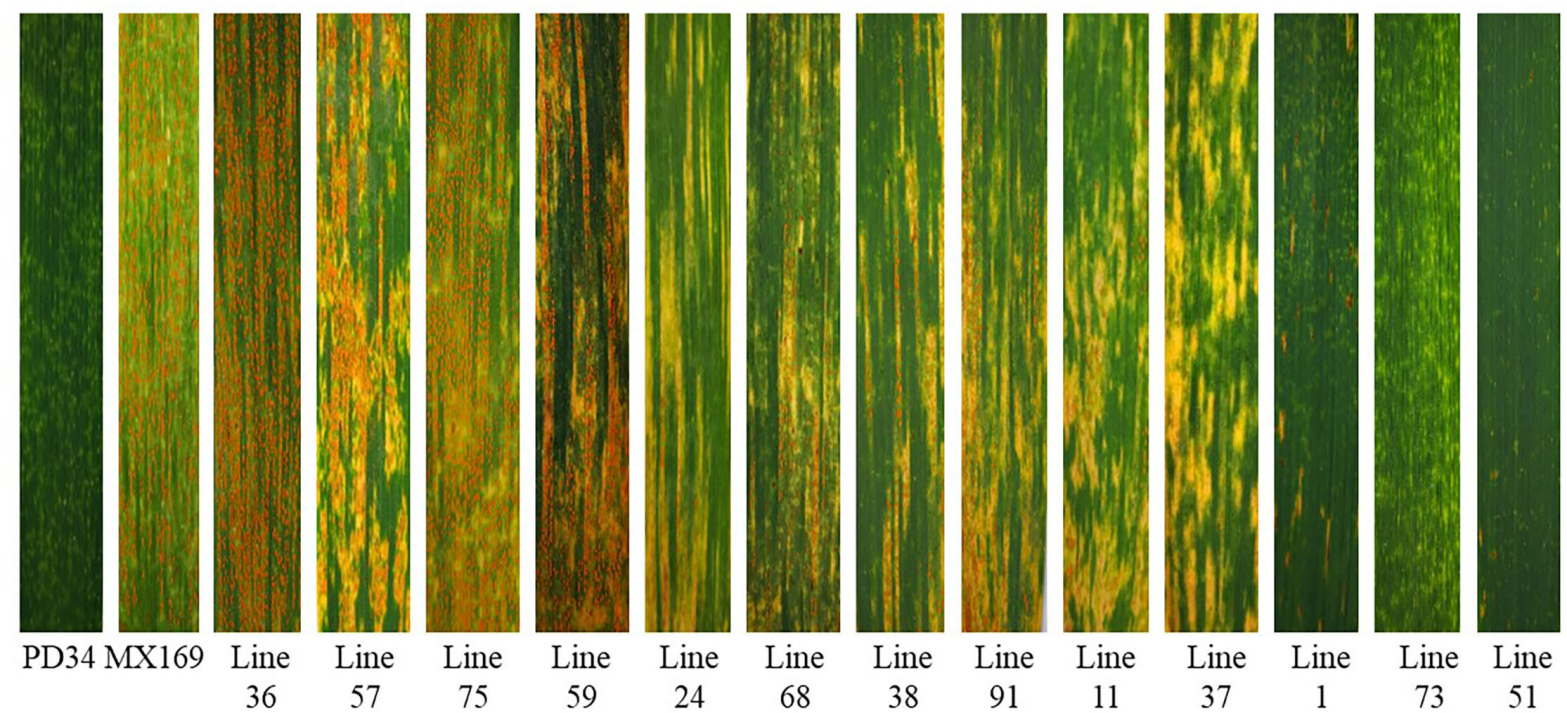
Stripe rust reactions on the leaves of resistant parent Pindong 34 (PD), susceptible parent Mingxian 169 (MX), and representative stripe rust reactions of some of the recombinant inbred lines (RILs).

A test of the 119 RIL populations from the Mingxian169 × Pingdong 34 cross at the seedling stage with the race CYR34 showed that Pindong 34 was highly resistant (IT 0) and Minxian 169 was susceptible (IT 9). The 119 F_6:10_ RIL population showed two-stage differentiation segregation in terms of IT, and the lines were classified into resistant (IT 0–2) and susceptible (IT 9) groups (Figures 1, 2). The ratio of resistant and susceptible families was 15:104 (expected ratio of 17:102), suggesting that three genes (two dominant and one recessive, χ^2^ = 0.21, *P* = 0.65) are responsible for resistance to race CYR34 in Pindong 34 at the seedling stage. In the greenhouse test, all the RILs were highly resistant (IT 0–2) or highly susceptible (IT 9), indicating that the race-specific all-stage resistance was mainly qualitative. Thus, the results showed that some genes conferred race-specific all-stage resistance in Pindong 34.

### Phenotypic Characterization of APR Resistance

**Figure 3:**
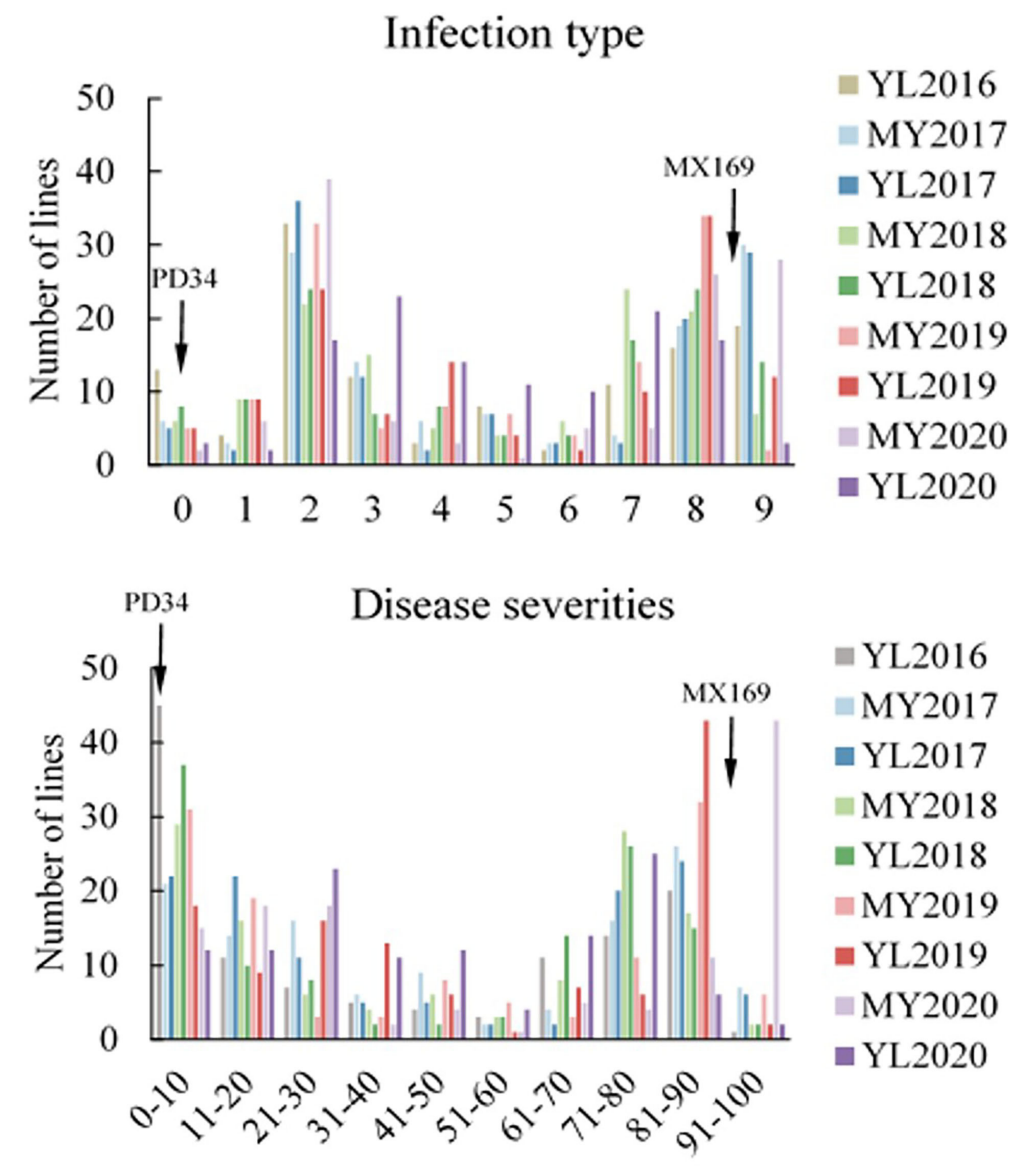
Frequency distributions of the mean infection type (IT) and disease severity (DS) for 119 RILs from the cross of Mingxian 169 (MX) × Pindong 34 (PD) grown at Mianyang (MY) and Yangling (YL) in 2016–2020. Arrows indicate the values of the parental lines.

Adult-plant resistance was evaluated in fields in Mianyang and Yangling from 2016 to 2020 under natural infection conditions. The susceptible parent Minxian 169 was susceptible (IT 9) at both seedling and adult-plant growth stages. The infection type of Pindong 34 was consistently 0 when tested at the adult-plant stage under field conditions, whereas Minxian 169 displayed 90–100% severity with IT=9 in all environments (Figure 3), indicating that stripe rust developed to levels adequate for collecting high-quality phenotypic data. The IT and DS values of the 119 individual RILs were continuously distributed from 0 to 9 and 0 to 100%, indicating that APR resistance in Pindong 34 was quantitatively inherited (Figure 3). In general, the ITs in Yangling were higher for most of the lines than the ITs in Mianyang (Figure 3). The correlation coefficients (r) between IT and DS were not very high between some of the 19 environments (r = 0.68–0.99, Table 1). The broad-sense heritability of both IT and DS was 0.97 (Table 2). Pairwise comparisons of the IT or DS values across years and within years were high, and all were significant (*P* < 0.001, Table 1). An ANOVA of IT and DS revealed significant differences (*P* < 0.01) in terms of the 119 RILs, 19 environments, and line × environment interactions (Table 2). No significant variations were detected among the replications within each experiment and the lines, suggesting that the expression of APR resistance was consistent across the different environments and over different years.

**Table 1:** Correlation coefficients (r) of the mean infection type (IT) and disease severity (DS) of the Mingxian 169 × Pindong 34-derived recombinant inbred lines tested in different environments.

	**IT**								**DS(%)**							
	**Mianyang**				**Yangling**				**Mianyang**				**Yangling**			
	**IT17MY**	**IT18MY**	**IT19MY**	**IT20MY**	**IT16YL**	**IT18YL**	**IT19YL**	**IT20YL**	**DS17MY**	**DS18MY**	**DS19MY**	**DS20MY**	**DS16YL**	**DS18YL**	**DS19YL**	**DS20YL**
17MY	1								1							
18MY	0.72 ***	1							0.60 ***	1						
19MY	0.76 ***	0.77 ***	1						0.69 ***	0.72 ***	1					
20MY	0.65 ***	0.66 ***	0.83 ***	1					0.58 ***	0.65 ***	0.80 ***	1				
16YL	0.77 ***	0.67 ***	0.61 ***	0.50 ***	1				0.72 ***	0.66 ***	0.60 ***	0.47 ***	1			
18YL	0.78 ***	0.73 ***	0.75 ***	0.63 ***	0.60 ***	1			0.78 ***	0.65 ***	0.74 ***	0.63 ***	0.66 ***	1		
19YL	0.74 ***	0.79 ***	0.87 ***	0.76 ***	0.58 ***	0.82 ***	1		0.70 ***	0.77 ***	0.82 ***	0.71 ***	0.61 ***	0.77 ***	1	
20YL	0.34 ***	0.30 **	0.37 ***	0.38 ***	0.33 ***	0.28 **	0.27 **	1	0.33 ***	0.31 **	0.38 ***	0.32 ***	0.30 **	0.28 **	0.23 *	1

Significance level at P < 0.05 (*), P < 0.01 (**), P < 0.001 (***).

**Table 2:** Analysis of variance and estimate of broad-sense heritability of the infection type (IT) and disease severity (DS) among 119 RILs from the cross of Mingxian 169 × Pindong 34 tested at Mianyang and Yangling in 2016–2020.

	**IT**				**DS**			
**Source of variation**	**Df**	**Mean square**	**F value**	***P* value**	**Df**	**Mean square**	**F value**	***P* value**
**Lines**	118	80.8	60.99	***	118	4695.36	59.76	***
**Blocks (environments)**	8	6.18	4*.66*	***	8	663.14	7.50	***
**Environments**	7	31.45	23.73	***	7	11785.82	149.99	***
**Lines** **× Environments**	772	5.84	4.41	***	772	400.40	5.10	***
**Error**	874	1.32			867	78.58		
^**a**^ ${\text{σ}}_{\text{g}}^{2}$	5.31				309.87			
^**b**^h^2^ (broad sense heritability)	0.93				0.92			

a *${\text{σ}}_{\text{g}}^{2}$ was estimated for genotypic (line) variances*.

b *h^2^ indicates the estimated heritability in the broad sense on the basis of the mean across replications and environments (or heritability per mean)*.

Significance level at P < 0.05 (*), P < 0.01 (**), P < 0.001 (***).

### Genetic Linkage Map Construction

A total of 11,346 SNP markers were used for linkage map construction, giving a total map length of 18,228.5 cM, with individual chromosomes ranging from 572.6 cM for chromosome 2B to 1,382.1 cM for chromosome 5B (Table 3). The number of markers per chromosome ranged from 76 for chromosome 4D to 342 for chromosome 5B, with an average number of 200 SNP markers. The average distance between neighboring SNP markers ranged from 2.5 cM/marker for chromosome 6A to 8.8 cM/marker for chromosome 7D, with an average number of 4.3 cM/marker. The map was used to identify significant associations between SNP markers and stripe rust resistance.

**Table 3:** Summary of chromosome assignment, number of SNP marker, map length, and marker density of the SNP genetic map of the 119 RILs from the cross of Mingxian 169 × Pindong 34.

**Chromosome**	**No. of SNP markers**	**Map length in cM**	**SNP intervals in cM**
1A	129	665.2	5.2
1B	156	572.6	3.7
1D	101	741.3	7.3
2A	245	1,046.4	4.3
2B	339	1,227.8	3.6
2D	218	893.5	4.1
3A	186	749.1	4
3B	240	877.3	3.7
3D	102	536.3	5.3
4A	155	958.3	6.2
4B	172	622.2	3.6
4D	76	611.1	8
5A	290	918.3	3.2
5B	342	1,382.1	4
5D	135	865.2	6.4
6A	304	765.3	2.5
6B	242	1,017.3	4.2
6D	176	857.4	4.9
7A	288	1,013	3.5
7B	176	785.1	4.5
7D	128	1,123.7	8.8
Total	4,200	18,228.5	-
Average	200	868.0	4.3

### QTL for ASR to Stripe Rust Determined Using the Greenhouse Seedling Data

**Figure 4:**
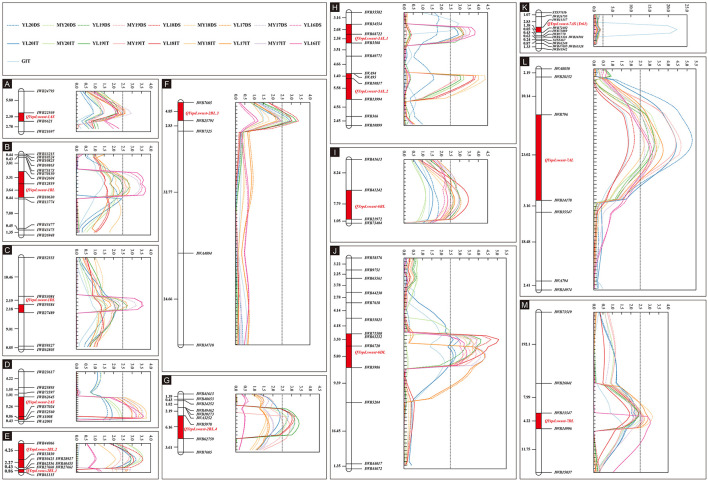
Stripe rust resistance QTLs on the genetic map of chromosomes 1AS, 1BL, 2AS, 2BL, 3AL, 6BL, 6DL, 7AS, 7AL, and 7DL based on IT and DS data **(A–M)**. The y-axis is in centimorgan distance (cM), and all genetic distance values shown are in the same scale; the x-axis indicates the LOD value, and all LOD values of QTL showed are in same scale except in K. The dotted line with different colors represents LOD value calculated based on the DS data from nine different environments. The solid line with different colors represents the LOD value calculated based on IT data from 10 different environments including greenhouse. The red rectangle on the genetic map indicates the corresponding QTL region.

The IT data of the 119 RILs tested with race CYR34 were used to identify QTLs for ASR. A total of eight markers representing four QTLs contributed by Pindong 34 were significantly associated with ASR, and one QTL each on 2AS, 2BL, and 7AS explained 9.69–13.58%, 9.49–12.07%, and 16.16% of the phenotypic variations in the tests with CYR34, respectively (Figure 4, Tables 4, 5). The lines without these QTLs were susceptible at the seedling stage and resistant at the adult-plant stage. The QTL *QYrpd.swust*-*2AS*, which was represented by the two closest flanking markers (*IWB62645* and *IWB52560*) in an interval of 6.1 cM, explained 9.69–13.58% of the phenotypic variation. The QTL *QYrpd.swust-2BL.4*, which was flanked by markers *IWB5978* and *IWB62759* on chromosome 2BL with a genetic distance of 6.1 cM, explained 9.86-12.07% and 9.49-11.59% of the phenotypic variation in IT and DS, respectively. In the greenhouse test, *QYrpd.swust*-*7AS* was represented by the two closest flanking markers (*IWB11337* and *IWB72402*) at a position of 2.6 cM on 7AS (Table 4). These three QTLs were effective against the tested race CYR34 and consistent with those determined through the ICIM-MET analysis of the RILs based on their IT for the race tests (Table 4).

**Table 4:** Summary of 15 stripe rust resistance QTLs identified using ICIM based on the mean disease severity (DS) and infection type (IT) of the 119 RILs from the cross of Mingxian 169 × Pindong 34 tested in Mianyang and Yangling in 2016–2020.

**QTL**	**Environment**	**Marker interval**	**IT**			**DS**		
			**^**a**^LOD**	**^**b**^PVE (%)**	**^**c**^Add**	**LOD**	**PVE (%)**	**Add**
*QYrpd.swust-1AS*	MY17	IWB22569 IWB6621	-	-	-	2.96	12.72	−11.31
	YL17		-	-	-	2.62	11.08	−11.05
	MY18		2.64	11.05	−0.90	2.63	11.08	−10.34
	YL18		-	-	-	2.60	12.61	−10.62
*QYrpd.swust-1BL*	YL16	IWB42604 IWB10630	3.72	13.33	−1.18	3.54	12.6	−12.97
	MY17		2.53	9.26	−0.96	3.43	12.19	−12.32
	YL17		-	-	-	2.89	10.38	−11.78
	MY18		-	-	-	2.81	10.24	−10.97
	MY19		2.68	9.64	−0.92	2.57	9.24	−11.21
	YL19		-	-	-	2.85	10.28	−10.55
*QYrpd.swust-1DL*	YL16	IWB59384 IWB27489	3.54	12.64	−1.12	3.60	13.05	−12.80
	MY17		-	-	-	3.22	12.47	−11.76
	YL17		-	-	-	2.76	10.81	−11.33
	YL19		-	-	-	2.72	9.81	−10.45
*QYrpd.swust-2AS*	YL16	IWB62645 IWB52560	3.62	13.27	−1.137	3.26	11.72	−12.16
	MY17		3.22	11.48	−1.09	3.58	12.67	−12.74
	YL17		3.02	10.84	−1.06	3.38	12.01	−12.64
	MY18		3.78	13.58	−1.07	3.51	12.64	−12.23
	YL18		3.65	13.18	−1.13	3.40	12.39	−12.01
	MY19		3.05	10.91	−0.96	2.71	9.69	−11.31
	YL19		3.49	12.74	−1.08	3.25	11.92	−11.32
	GIT^d^		2.65	9.94	−0.81	-	-	-
*QYrpd.swust-2BL*.1	MY17	IWB27661 IWB61115	2.92	10.57	−1.01	2.67	9.70	−10.92
	YL17		2.77	10.03	−1.03	2.67	9.69	−11.19
	MY18		-	-	-	2.71	9.92	−10.71
*QYrpd.swust-2BL*.2	YL18	IWB44066 IWB30421	3.09	11.21	−1.07	3.58	12.97	−12.60
	MY19		2.85	10.36	−0.95	-	-	-
	YL19		-	-	-	2.94	10.57	−11.02
	MY20		3.58	12.76	−1.16	3.43	12.25	−13.83
	YL20		2.95	10.76	−0.77	3.21	11.72	−9.35
*QYrpd.swust-2BL*.3	MY17	IWB7605 IWB25791	2.72	10.11	−0.99	2.86	10.63	−11.29
	YL17		2.55	9.54	−1.00	2.64	9.87	−11.29
	YL18		3.20	11.98	−1.07	3.48	13.15	−12.28
	YL19		3.18	12.43	−1.05	2.95	11.61	−10.98
	MY20		2.65	11.83	−1.01	2.53	11.25	−11.84
	YL20		-	-	-	2.98	10.96	−8.95
*QYrpd.swust-2BL*.4	YL18	IWB5978 IWB62759	3.03	11.06	−1.06	3.18	11.59	−11.63
	MY19		2.92	10.69	−0.98	2.57	9.49	−11.42
	YL19		3.41	12.07	−1.12	3.18	11.44	−11.69
	MY20		2.71	9.86	−1.03	2.75	9.97	−12.38
	YL20		-	-	-	2.82	10.19	−8.58
	GIT^d^		3.18	11.52	−0.91	-	-	-
*QYrpd.swust-3AL*.1	YL16	IWB34554 IWB3508	3.83	13.83	−1.97	3.57	12.96	−12.89
	MY17		3.34	12.66	−1.07	3.26	13.21	−11.80
	YL17		3.22	12.05	−1.10	3.21	12.55	−12.15
	YL19		-	-	-	3.18	11.41	−11.26
	YL20		2.93	11.51	−0.76	-	-	-
	GIT^d^		2.96	14.80	−0.86	-	-	-
*QYrpd.swust-3AL*.2	MY18	IWA95 IWB13994	4.02	14.68	−1.10	4.37	16.10	−13.23
	YL18		3.73	13.67	−1.15	3.85	13.97	−12.93
	MY19		2.77	11.55	−0.93	2.89	12.41	−11.74
	YL19		3.06	13.02	−1.03	-	-	-
	MY20		3.22	12.67	−1.10	3.21	13.12	−13.19
*QYrpd.swust-6BL*	MY17	IWB41242 IWB21972	2.96	10.82	−1.08	3.00	10.86	−12.12
	YL17		2.74	9.9	−1.09	3.07	11.14	−12.68
	MY18		2.68	9.89	−0.97	-	-	-
	YL18		3.47	12.78	−1.17	2.96	10.92	−12.02
	MY19		2.65	9.95	−0.93	2.52	9.39	−11.50
	YL19		3.02	11.18	−1.06	2.65	9.93	−10.85
*QYrpd.swust-6DL*	YL16	IWB71500 IWA5986	3.39	12.06	−1.17	3.18	11.37	−12.79
	MY17		4.39	14.50	−1.32	4.13	14.17	−14.06
	YL17		4.36	14.42	−1.37	4.34	14.84	−14.92
	MY18		3.74	13.76	−1.11	3.39	12.79	−12.13
	YL18		5.10	17.76	−1.458	4.68	16.36	−15.41
	MY19		3.95	14.16	−1.18	3.67	13.33	−14.10
	YL19		4.14	15.06	−1.27	3.58	13.18	−12.83
	MY20		2.51	9.54	−1.01	2.51	9.52	−12.06
	YL20		2.66	9.72	−0.79	3.00	10.64	−9.76
*QYrpd.swust-7AS*	GIT^d^	IWB11337 IWB72402	22.41	16.16	−2.80	-	-	-
*QYrpd.swust-7AL*	YL16	IWB796 IWB14178	3.04	9.77	−1.50	3.15	10.49	−16.89
	MY17		3.31	11.06	−1.51	2.75	9.28	−15.25
	YL17		3.74	12.00	−1.71	3.37	11.07	−17.33
	MY18		2.72	9.09	−1.25	3.41	11.14	−16.50
	MY19		4.43	14.11	−1.57	4.70	14.64	−21.37
	YL19		3.25	11.29	−1.38	2.98	10.27	−14.47
	MY20		3.29	10.74	−1.53	3.52	11.42	−17*.00*
	YL20		4.44	14.84	−1.22	5.30	17*.00*	−15.17
*QYrpd.swust-7DL*	YL16	IWB13547 IWB14996	3.04	10.91	−1.09	2.59	9.57	−11.25
	MY17		2.66	9.34	−1.01	2.52	8.87	−10.97
	YL17		2.92	10.34	−1.09	2.73	9.64	−11.77
	MY18		2.66	9.50	−0.95	3.17	11.38	−11.57
	MY20		-	-	-	2.57	9.17	−12.10

a *LOD, logarithm of odds score*.

b *PVE, percentage of the phenotypic variance explained by individual QTLs*.

c *Add, additive effect of the resistance allele*.

d *GIT, greenhouse infection type*.

**Table 5:** Summary of 15 stripe rust resistance QTLs discovered using the MET for the RIL population from the cross of Mingxian 169 × Pindong 34 tested in Mianyang and Yangling in 2016–2020.

**Chr**	**Left Marker**	**Right Marker**	**LOD**	**LOD (A)**	**LOD (A×E)**	**PVE**	**PVE(A)**	**PVE (A×E)**
1A	IWB24739	IWB22569	13.66	6.10	7.57	3.03	1.54	1.48
1B	IWB12819	IWB10630	44.13	24.10	20.03	12.68	6.37	6.31
1D	IWB59384	IWB27489	14.31	3.76	10.55	3.99	0.93	3.06
2A	IWB62645	IWB37024	22.29	9.97	12.31	4.54	2.53	2.00
2B	IWB27661	IWB61115	13.17	6.12	7.06	3.14	1.53	1.61
2B	IWB44066	IWB13830	12.75	6.15	6.60	2.97	1.53	1.44
2B	IWB25791	IWB7325	29.58	16.35	13.23	8.26	4.17	4.09
2B	IWB5978	IWB62759	12.17	2.75	9.41	1.87	0.70	1.18
3A	IWB68722	IWB3508	11.81	2.18	9.63	1.29	0.55	0.74
3A	IWB10817	IWB13994	41.30	23.88	17.42	12.85	6.31	6.54
6B	IWB43467	IWB41242	2.94	0.49	2.46	0.47	0.12	0.35
6D	IWB65561	IWB44230	11.48	5.88	5.60	2.91	1.48	1.44
7A	IWB63232	IWB16720	68.99	35.70	33.28	19.38	9.73	9.65
7A	IWB796	IWB14178	33.90	17.20	16.70	8.54	4.35	4.19
7D	IWB26841	IWB13547	27.00	14.70	12.3	7.28	3.72	3.56

### QTL for Stripe Rust Resistance Determined Using the Field Adult-Plant Data

Because the RILs had various IT and DS values that did not form distinct resistance and susceptible classes, the IT and DS data of the 19 field experiments were used for QTL mapping. Inclusive composite interval mapping (ICIM) identified 12 APR QTLs on chromosome arms 1AS, 1BL, 1DL, 2AS, 2BL, 3AL, 6BL, 6DL, 7AL, and 7DL (Figure 4). All alleles for *Pst* resistance of the QTL located on chromosome arms were derived from Pindong 34 and designated as *QYrpd.swust-1AS, QYrpd.swust-1BL, QYrpd.swust-1DL, QYrpd.swust-2BL.1, QYrpd.swust-2BL.2, QYrpd.swust-2BL.3, QYrpd.swust*-*3AL.1, QYrpd.swust-3AL.2, QYrpd.swust-6BL, QYrpd.swust-6DL, QYrpd.swust-7AL*, and *QYrpd.swust-7DL*, and they were significant in some of the tests for both IT and DS (*P* < 0.005, LOD > 2.5). The lines containing these QTLs were susceptible to stripe rust at the seedling stage and resistant at the adult-plant stage. Table 4 presents a summary of the IT and DS effects of the individual QTLs across the 2016–2020 seasons in this study.

Among the 12 QTLs detected for APR resistance, *QYrpd.swust-1BL, QYrpd.swust*-*3AL.1, QYrpd.swust-3AL.2, QYrpd.swust-6BL, QYrpd.swust-6DL, QYrpd.swust-7AL*, and *QYrpd.swust-7DL* were significantly more abundant than in the five of the tested environments in terms of both IT and DS (*P* < 0.05, LOD > 2.5) (Tables 4, 5). *QYrpd.swust-1BL* explained an average of 9.24–13.33% of the phenotypic variation in IT and DS across the six environments of Mianyang and Yangling. This QTL was located within 7.0 cM between markers *IWB42604* and *IWB10630* on the long arm of chromosome 1B. *QYrpd.swust*-*3AL.1* was represented by the two closest flanking markers (*IWB34554* and *IWB3508*) at a genetic distance of 5.1 cM on 3AL, and it explained 11.41–14.80% of the IT and DS phenotypic variation in five environments. *QYrpd.swust-3AL.2* was located in an interval of 7 cM between *IWA95* and *IWB13994* on chromosome 3AL, and it explained 11.54–17.0% of the IT and DS phenotypic variation in five environments. *QYrpd.swust-6BL*, which was flanked by markers *IWB41242* and *IWB21972* on chromosome 6BL with a genetic distance of 7.8 cM, explained 9.39–12.78% of the phenotypic variation in IT and DS in one of the six environments. *QYrpd.swust-6DL* explained 9.52% and 16.36% of the phenotypic variation of IT and DS on average, respectively, across nine environments. This QTL was located within a 9.1 cM interval between markers *IWB71500* and *IWA5986* on the long arm of chromosome 6D. *QYrpd.swust-7AL* explained 9.09–17.0% of the phenotypic variance depending upon the experiment, and the closest markers were *IWB796* and *IWA5986*, with a genetic distance of 22.0 cM in eight environments. *QYrpd.swust-7DL* was flanked by markers *IWB13547* and *IWB14996* on chromosome 7DL with a genetic distance of 4.2 cM, and it explained 8.87–11.38% of the phenotypic variation in IT and DS in five environments (Table 4, Figure 4).

The remaining QTLs, including *QYrpd.swust-1AS, QYrpd.swust-1DL, QYrpd.swust-2BL.1, QYrpd.swust-2BL.2*, and *QYrpd.swust-2BL.3*, were significant in at least three environments and explained 11.05–12.72%, 9.81–13.05, 9.69–10.57%, 10.36–12.97%, and 9.54–13.15% of the phenotypic variation, respectively (Table 5). *QYrpd.swust-1AS* was located in an interval of 2.3 cM and flanked by markers *IWB22569* and *IWB6621* on chromosome 1AS, and *QYrpd.swust-1DL* was located in an interval of 2.2 cM and flanked markers *IWB59384* and *IWB27489* on chromosome 1DL. *QYrpd.swust-2BL.1, QYrpd.swust-2BL.2*, and *QYrpd.swust-2BL.3* were located in three intervals of 0.9 cM, 6.5 cM, and 2.8 cM and flanked by markers *IWB27661*–*IWB61115, IWB44066*–*IWB30421*, and *IWB7605*–*IWB25791*, respectively. These three QTLs were present on the long arm of the 2B chromosome.

### Digenic Epistatic QTL

The ICIM-EPI scan for digenic epistatic QTLs within the MET function yielded 48 marker pairs with LOD scores > 10 and 22 pairs with LOD scores > 20. Only two of the 2 × 15 markers were located near a monogenic QTL (Table 6).

**Table 6:** Multi-environmental analysis and digenic epistatic QTLs based on an ICIM-EPI analysis of the mean infection type (IT) and disease severity (DS) of the RILs from the cross of Mingxian 169 × Pindong 34 tested in Mianyang and Yangling in 2016–2020.

**Chromosome1**	**Position1**	**Left marker1**	**Right marker1**	**Chromosome2**	**Position2**	**Left marker2**	**Right marker2**	**LOD**
1B	*0*	*IWB10823*	*IWB69863*	1B	10	*IWB12819*	*IWB10630*	18.59
1B	*0*	*IWB10823*	*IWB69863*	1D	15	*IWB27489*	*IWB59327*	28.01
2B	*5*	*IWB25791*	*IWB7325*	2B	35	*IWB7325*	*IWA4894*	16.18
1A	*10*	*IWB6621*	*IWB21697*	3A	10	*IWB3508*	*IWB40771*	16.26
2A	*5*	*IWB25893*	*IWB73597*	6B	0	*IWB43467*	*IWB41242*	27.06
2A	*5*	*IWB25893*	*IWB73597*	6D	20	*IWB55825*	*IWB71500*	15.20
2B	*30*	*IWB7325*	*IWA4894*	6D	40	*IWA5204*	*IWB44017*	16.16
2B	*30*	*IWB7325*	*IWA4894*	7A	5	*IWB72402*	*IWB73889*	18.06
6B	*0*	*IWB43467*	*IWB41242*	7A	55	*IWB35547*	*IWA794*	20.53
6D	*55*	*IWA5204*	*IWB44017*	7A	55	*IWB35547*	*IWA794*	36.15
3A	*30*	*IWB366*	*IWB10899*	7D	0	*IWB73519*	*IWB26841*	15.77
6B	*5*	*IWB43467*	*IWB41242*	7D	30	*IWB13547*	*IWB14996*	17.17
6D	*55*	*IWA5204*	*IWB44017*	7D	35	*IWB14996*	*IWB15037*	17.72

### Additive Interactions Between Detected Resistance Loci

**Figure 5:**
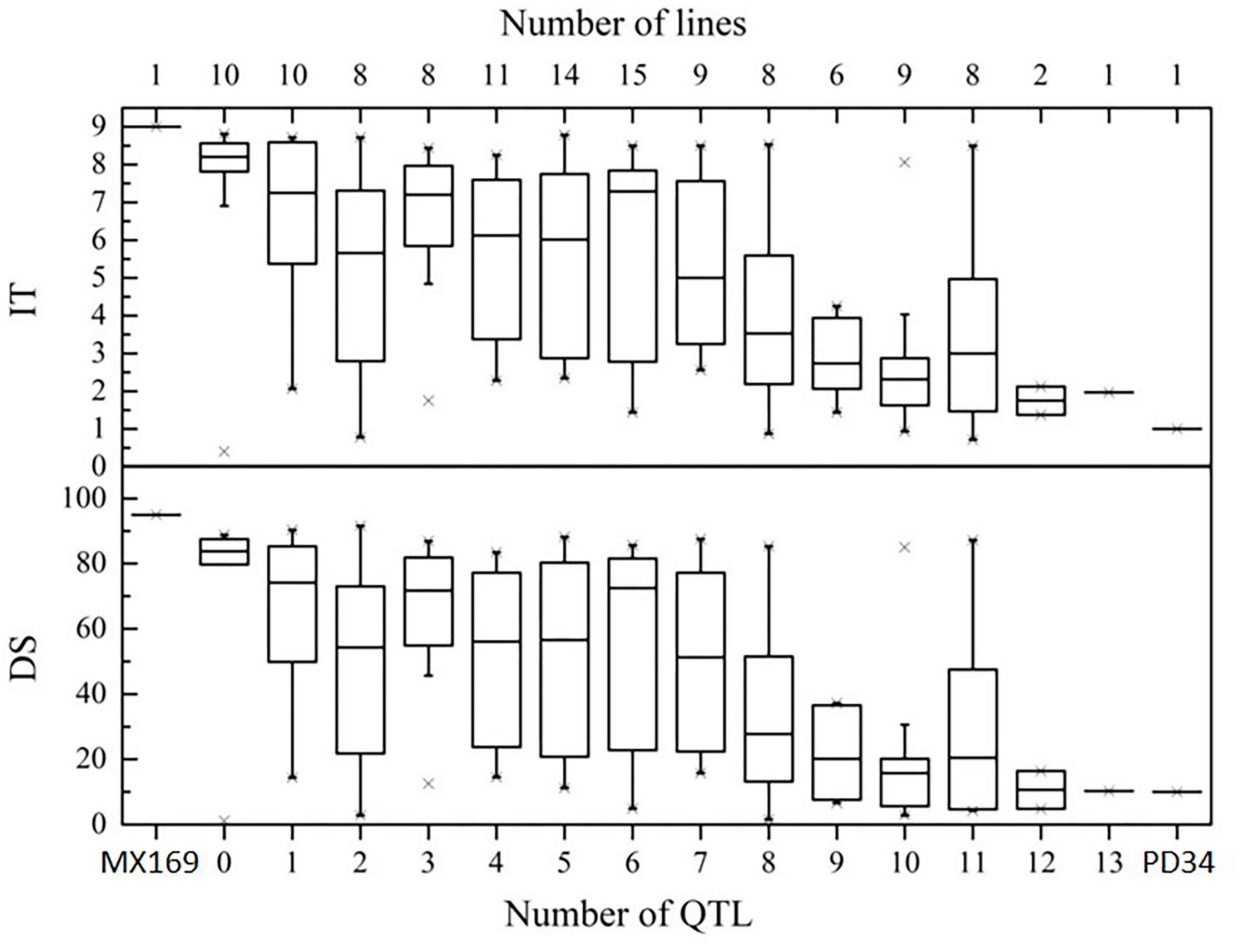
Effects of individual QTLs and their combinations on stripe rust scores illustrated by the mean infection type (IT) and disease severity (DS) scores of the RILs from the cross of Mingxian 169 × Pindong 34 in combined environments (MY and YL). The box plots indicate the infection type (IT) and disease severity (DS) associated with the identified QTL and their combination. The resistance alleles present in the group are indicated by the name of the chromosome where the QTL is located. The number of RILs grouped in each class is indicated in parentheses by each class name. Error bars represent ± 1 SEM.

To determine the effects of QTLs in various combinations for *Pst* resistance, the 119 RILs were classified into genotypic groups based on the presence of markers closely associated with the 15 QTLs (Table 4). These genotypes were further sorted into 13 groups based on the number of potential QTLs for *Pst* resistance. Figure 5 shows the differences in the mean IT and DS values of the 13 groups. In general, there were significant additive effects for stripe rust resistance in some RIL lines, and all QTL combinations were significantly different (*P* < 0.05) from the RILs with no resistance alleles. When a few QTLs were combined, the average IT and DS values were lower than those with only one or none (Figure 5), which is supported by the significant additive effects (*P* < 0.01) obtained by the QTL analysis.

## Discussion

The wheat cultivar Pindong 34 has maintained good resistance to stripe rust under field conditions since it was initially bred in 1990. In 2014, Zhou identified and performed molecular mapping of its all-stage resistance gene *Yr61* by employing bulk segregant analysis in combination with the resistance gene analog polymorphism (RGAP), sequence-tagged site (STS), and simple sequence repeat (SSR) genotyping methods in the F_2_ and F_2:3_ populations. In this study, a 90K SNP-chip genotyping assay and RIL population containing 119 lines were used to identify and analyze all-stage and adult-plant resistance, and a total of 12 QTLs for APR resistance together with 3 QTLs for all-stage resistance were obtained on 10 different chromosomes encompassing 15 regions. Compared to previous studies that performed QTL mapping, the results showed the highest number of QTLs for *Pst* resistance in a single wheat cultivar, which also explained why the cultivar Pingdong 34 could maintain a high level of field resistance for the long term.

### Comparison With Previously Mapped Resistance Genes and QTLs

#### QYrpd.Swust-1AS

*QYrpd.swust-1AS* is derived from Pingdong 34, and it was flanked by markers *IWB22569* and *IWB6621* and corresponds to the 1,174,937–3,256,249 bp region on chromosome 1AS of CS. It explained 11.05–12.72% of the phenotypic variation in five experimental environments. To date, some genes/quantitative trait loci (QTL) regions have been reported for stripe rust resistance on chromosome 1AS, and five of them were located on the short arm of chromosome 1AS: *YrTtd-1AS* (Liu et al., 2017), *QYr.tam-1A.1* (Basnet et al., 2014a), *QYrus.vt-1AS* (Christopher et al., 2013), *QYrMa.wgp-1AS* (Liu et al., 2018), and *QYr.cim-1AS* (Lan et al., 2015). The gene *YrTtd-1AS* and QTLs *QYrMa.wgp-1AS* and *QYr.cim-1AS* are all-stage resistance (ASR) genes; therefore, they cannot be identical to *QYrpd.swust-1AS*, which is an adult-plant resistance gene (Lan et al., 2015; Liu et al., 2017, 2018). *QYr.tam-1A.1* confers APR resistance against stripe rust, although it is a minor QTL (Basnet et al., 2014a). Based on the relative marker positions, the QTL *QYrpd.swust-1AS* in the current study differs from the resistance genes previously identified.

#### QYrpd.Swust-1BL

*QYrpd.swust-1BL* was located between markers *IWB42604* and *IWB10630* on the long arm of chromosome 1B and was mapped to the 520,810,752 bp−520,926,056 bp region of chromosome 1BL of the CS genome. To date, the number of QTLs/genes for stripe rust resistance on chromosome 1BL has been reported to be more than 10 based on the genetic populations and GWAS analysis, and they include *YrChk* (Liu et al., 2007), *YrExp1* (Lin and Chen, 2008), *YrP138* (Yue et al., 2010), *YrTtd-1BL* (Liu et al., 2017), QYr.cimmyt-1BL (William et al., 2006), and *YrN.S-2* (Li et al., 2010), which confer all-stage resistance, and *QYrPI181410.wgp-1BL* (Liu et al., 2020), *QYrdr.wgp-1BL.1* (Hou et al., 2015), *QYrdr.wgp-1BL.2* (Hou et al., 2015), and *QYrex.wgp-1BL* (Lin and Chen, 2009), which confer HTAP resistance. The QTL *QYrpd.swust-1BL* identified in the present study confers adult resistance. Further study is needed to confirm whether the resistance of QYrpd.swust-1BL is dependent on temperature and to investigate the relationships between QYrpd.swust-1BL and the above HTAP genes. *Yr29* (William et al., 2006), *QYr.tam-1B* (Basnet et al., 2014a), *QYr.cim-1BL* (Lan et al., 2014), *QYrTtd-1BL* (Liu et al., 2017), *QPst.jic-1B* (Melichar et al., 2008), and *QYr.ucw-1BL* (Cobo et al., 2018) all confer APR resistance. *Yr29*, a permanently designated gene for resistance to wheat stripe rust in wheat cultivar 'Pavon76,' was linked with SSR markers Xwmc44 and Xwmc367 and located at the distal end of chromosome 1BL. *QYr.tam-1B*, a QTL derived from the common wheat cultivar ‘Quaiu 3,' was linked with the markers *wPt-668027* and *cSLV46G22*. *QYr.cim-1BL*, derived from wheat Francolin#1, was mapped between markers *csLV46* and *wPt-9028*. *QYrTtd-1BL*, a QTL for resistance to wheat stripe rust in emmer wheat (*Triticum turgidum* ssp. *dicoccum*) was flanked by SNP marker *IWB69464*. *QPst.jic-1B*, a QTL derived from UK winter wheat cultivar Guardian, was flanked with the SSR markers *Xgwm259* and *Xgwm818*. *QYr.ucw-1BL* was mapped to the SNP marker *IWA8581* and derived from the wheat cultivar Klein Chajá. *QYrsv.swust-1BL.1* was mapped within a 0.75-cM region in *T*. *turgidum* subsp. *durum* 'Svevo' on chromosome lBL, corresponding to the region of 670.7 to 671.5 Mb on the Chinese Spring chromosome 1BL. According to the resistance types and the physical location of molecular markers, it can be preliminarily inferred that the APR QTL *QYrpd.swust-1BL* identified in this study is different from the APR QTLs identified in previous studies.

#### QYrpd.Swust-1DL

*QYrpd.swust-1DL* was flanked by *IWB59384* and *IWB27489* and explained 9.81–13.05% of the phenotypic variation in four environments. The *QYrpd.swust-1DL* region was located in the physical region of 378,867,221 bp−379,141,846 bp. The permanently designated gene *Yr25* was reported on chromosome 1D in some *Pst* races in other countries, although it has been largely ineffective in the *Pst* population in the US, and its specific chromosomal location is unknown (Calonnec and Johnson, 1998). There are some temporarily named genes for stripe rust reported on 1DL, e.g., *QYrww.wgp.1D-3*, a QTL for resistance to wheat stripe rust flanked by SNP marker *IWA3446* and physically located at approximately 2,331,833 bp on 1DL, which differed from the location of *QYrpd.swust-1DL* (Tian et al., 2011).

#### QYrpd.Swust-2AS

*QYrpd.swust-2AS* was detected for all-stage resistance and flanked by *IWB62645* and *IWB52560*. It explained 9.69–13.58% of the phenotypic variation in eight environments. Officially and temporarily designated *Yr* genes for resistance to wheat stripe rust reported on 2AS included *Yr17* (Jia et al., 2011) and *YrR61* (Hao et al., 2011). *Yr17* from *Aegilops tauschii* conferred seedling (all-stage) resistance and was mapped to the distal end of chromosome arm 2AS, with the closest SSR markers *Xgwm636* and *Xgwm359* (Jia et al., 2011). *YrR61*, derived from soft red winter wheat Pioneer 26R61, was flanked by markers *Xbarc124* and *Xgwm359*, and assigned to the distal 22% of the short arm of wheat chromosome 2A (Hao et al., 2011), such as *QYrTtd-2AS* (Liu et al., 2017), *QYrww.wgp.2A-1* (Mu et al., 2020), *Qyr.wpg-2A.2, Qyr.wpg-2A.3*, and *Qyr.wpg-2A.4* (Naruoka et al., 2015), while APR genes on the short arm of chromosome 2A included *YrSph* (Chen et al., 2012), *QYrPI182103.wgp-2AS* (Feng et al., 2018), and *QYrMa.wgp-2AS* (Liu et al., 2018); therefore, these QTLs cannot be identical to *QYrpd.swust-2AS*. On the short arm of 2A, most QTLs that confer ASR were in the distal region of *QYrpd.swust-2AS*, such as *Yr69* (Hou et al., 2016) and *YrSph* (Chen et al., 2012). *Yr56* confers ASR in the wheat cultivar Wollaroi (AUS99174) and was flanked by the closed SSR markers *Xsun167* and *Xsun168* (Bansal et al., 2014). *QYrMa.wgp-2AS* confers ASR in the winter wheat cultivar Madsen, and it was flanked with the closed SNP marker *IWB35714*. Based on the origin, disease resistance type, and linked markers, *QYrzv.swust-2AS* is unlikely to represent the previously reported QTL.

#### QYrpd.Swust-2BL.1, QYrpd.Swust-2BL.2, QYrpd.Swust-2BL.3, and QYrpd.Swust-2BL.4

Three APR resistance QTLs, *QYrpd.swust-2BL.1, QYrpd.swust-2BL.2*, and *QYrpd.swust-2BL.3*, and one ASR QTL, *QYrpd.swust-2BL.4*, were identified on the long arm of chromosome 2B. *QYrpd.swust-2BL.1* was located between markers *IWB27661* and *IWB61115* and mapped to the 773,785,836 bp−775,169,437 bp region of the CS genome on chromosome 2BL. *QYrpd.swust-2BL.2* was located between markers *IWB44066* and *IWB30421* and mapped to the 775,368,259 bp−777,519,972 bp region of the CS genome on chromosome 2BL. *QYrpd.swust-2BL.3* was located between markers *IWB7605* and *IWB25791* and mapped to the 793,151,400 bp−797,995,314 bp region of the CS genome on chromosome 2BL. *QYrpd.swust-2BL.4* was located between markers *IWB5978* and *IWB62759* and mapped to the 782,534,687 bp−784,551,415 bp region of the CS genome on chromosome 2BL. To date, more than 20 genes and QTLs have been reported on chromosome 2BL based on genetic populations and GWAS analysis. *QYr.caas-2BL.2* (Ren et al., 2015), *QYrPI181410.wgp-2BL* (Liu et al., 2020), and *QYrdr.wgp-2BL* (Hou et al., 2015) code for completely different types of resistance relative to the four QTLs in the present study. Based on the physical position in the durum genome, some genes and QTL loci are completely different from the *QYrpd.swust-2BL.1, QYrpd.swust-2BL.2, QYrpd.swust-2BL.3*, and *QYrpd.swust-2BL.4* loci. These include *Yr7* (Macer, 1963), *Yr43* (Cheng and Chen, 2010), *Yr44* (Sui et al., 2009), *Yr53* (Xu et al., 2013), *YrQz* (Deng et al., 2004), *YrSP* (Feng et al., 2015b), *YrSte* (Chen et al., 1996), *YrV23* (*Yr3a*) (Wang et al., 2006), *YrTtd-2BL.1* and *YrTtd-2BL.2* (Liu et al., 2017), *QYr-2B* (Boukhatem et al., 2002), *QYr.tam-2BL* (Basnet et al., 2014b), *QYr.nafu-2BL* (Zhou et al., 2015), *QYrns.orz-2BL* (Vazquez et al., 2015), *QYrww.wgp.2B-4* (Mu et al., 2020), *QYrdr.wgp-2BL* (Hou et al., 2015), *QYr.csiro-2BL* (Rosewarne et al., 2008), *QYR1* (Boukhatem et al., 2002), *QYr.inra-2BL* (Mallard et al., 2005), *QYrcaas-2BL* (Ren et al., 2012), and *QYraq.cau-2BL* (Ramburan et al., 2004).

#### QYrpd.Swust-3AL.1 and QYrpd.Swust-3AL.2

Two QTLs were identified on the long arm of chromosome 3A. *QYrpd.swust-3AL.1* was located between markers *IWB34554* and *IWB3508* and mapped to 731,222,152 bp−732,779,714 bp of the CS chromosome 3AL, and it confers APR resistance. *QYrpd.swust-3AL.2* was located between markers *IWA95* and *IWB13994* and mapped to the 724,201,099 bp−725,738,951 bp of CS chromosome 3AL, and it confers APR resistance. The two QTLs *YrTr2* (Chen and Line, 1992) and *QYrne.vt-3A* (Christopher et al., 2013) were mapped to chromosome 3A, although their specific chromosomal location is unknown. Other QTLs are mapped on the long arm of chromosome 3A, such as *QYrdr.wpg-3AL*, which confers HTAP resistance derived from the winter wheat cultivar Druchamp (Hou et al., 2015), are likely different from *QYrpd.swust-3AL.1* and *QYrpd.swust-3AL.2*. *QYrPI182103.wgp-3AL* (Feng et al., 2018), *YrTtd-3AL* (Liu et al., 2017), and *QYrww.wgp-3A* (Mu et al., 2020) confer all-stage resistance, and *QYrPI182103.wgp-3AL* was linked with markers *IWA899* and *Xgwm2* and derived from spring wheat PI 182103. *QTL YrTtd-3AL* was linked with SNP marker *IWB71901* and derived from emmer wheat (*Triticum turgidum* ssp. *dicoccum*). QTL *QYrww.wgp.3A* was linked with marker *IWB44443* (Mu et al., 2020), and QTL *QYrst.orr-3AL* was linked with marker *wPt1652* and derived from spring wheat Stephens (Vazquez et al., 2012). Based on the disease resistance type and linked markers, *QYrpd.swust-3AL.1* and *QYrpd.swust-3AL.2* are unlikely to be the above previously reported QTLs.

#### QYrpd.Swust-6BL

*QYrpd.swust-6BL* was located between markers *IWB41242* and *IWB21972* on the long arm of chromosome 6B and mapped to the 423,190,456 bp−651,418,439 bp region of the CS genome on chromosome 6BL, and it confers APR resistance. To date, more than nine QTLs/genes for stripe rust resistance have been reported on chromosome 6BL by genetic populations and GWAS analysis. For example, *YrLM168a* (derived from wheat resistance line LM168a, nearest flanking markers *Xwmc756* and *Xbarc146*, Feng et al., 2015a) confers all-stage resistance and *QYrdr.wgp-6BL.2* (derived from winter wheat Druchamp, most closely associated with SNP marker *IWA6420*) confers HTAP resistance (Hou et al., 2015). *QYrdurum-6BL* was derived from durum wheat and was linked with the SNP marker *IWB72946* (Liu et al., 2017). The QTL derived from wheat cultivar Pastor conferred APR resistance and was mapped between markers *XwPt-6329* and *XwPt-5176* on chromosome 7BL (Rosewarne et al., 2012). *QYrww.wgp.6B* was linked with the SNP marker *IWA1017* (Mu et al., 2020), and *QYr.tam-6B* originated from the wheat cultivar Quaiu and was linked with markers *wpt-0171* and *wpt-4164* (Basnet et al., 2014b). *QYr.wsu-6B.2* and *QYr.wsu-6B.3* (Bulli et al., 2016) were linked by *XIWA4169* and *XIWA349*, respectively. The two QTLs *Qyr.wpg-6B.1* and *Qyr.wpg-6B.2* were detected on the long arm of 6B and linked with markers *IWA7257* and *IWA3222*, respectively (Naruoka et al., 2015). *QYr.ucw-6B_IWA7257* was identified with the linked marker *XIWA7257* (Maccaferri et al., 2015). Based on the linked marker origins and disease resistance type, *QYrpd.swust-6BL* is unlikely to be the above previously reported QTLs.

#### QYrpd.Swust-6DL

*QYrpd.swust-6DL* was flanked by *IWB71500* and *IWA5986* on chromosome 6DL with a genetic distance of 9.1 cM, and it explained 9.52–16.36% of the phenotypic variation in nine environments in the BIP analysis and conferred APR resistance. The *QYrpd.swust-6DL* region was located in the 576,040,835 bp−72,231,237 bp region of the CS 6DL chromosome. The two genes *Yr20* (Chen et al., 1995a,b) and *Yr23* (Chen et al., 1995a,b) were also mapped on chromosome 6DL and conferred ASR, which was different from the resistance type of *QYrpd.swust-6DL*. Other QTLs mapped on the long arm of chromosome 6D, such as *YrH9020a* (flanking markers *Xbarc202* and *Xbarc96*), which conferred APR resistance, were derived from wild relative *P. huashanica* H9020-1-6-8-3 (Liu et al., 2014). *QYr.sicau-6D* was linked with marker *Xcfd95-2*. *QYr.ufs-6D* was mapped based on the SSR markers *Xgwm325* and *Xbarc175* and is located in the 79,956,573 bp−79,956,710 bp region of the CS 6DL chromosome. *QYr.sicau-6D* overlapped with *QYr.ufs-6D* on chromosome 6D, and both the two QTLs conferred APR resistance (Agenbag et al., 2012; Yao et al., 2019). Based on the resistance type and linkage markers, *QYrpd.swust-6DL* is probably different from the above QTLs.

#### QYrpd.Swust-7AS

A QTL that conferred ASR was identified on the short arm of chromosome 7A. *QYrpd.swust-7AS* was located between markers *IWB11337* and *IWB72402* and mapped to the 18,877,040 bp−20,172,969 bp region of the CS genome on the chromosome 7AS. To date, only one formally named *Yr* gene, *Yr61*, has been reported on chromosome 7AS, and it confers all-stage resistance. *Yr61* originated from the winter wheat cultivar Pindong 34, conferred all-stage resistance, and was located between STS markers *Xwgp5765b* and *Xwgp5467* on the short arm of 7A (Zhou et al., 2014). The two STS markers could also be detected in the lines containing the QTL *QYrpd.swust-7AS*. YrTtd-7AS^*^ was linked with SNP marker *IWB61392* and derived from the Chinese cultivar emmer wheat (*Triticum turgidum* ssp. *dicoccum*) (Liu et al., 2017), and it was mapped to the 14,814,722 bp−14,814,722 bp region. Other previously reported QTLs, such as *QYr.caas-7AS* (Ren et al., 2012), *QYrdurum-7AS.1, QYrdurum-7AS.2, QYrdurum-7AS.3* (Liu et al., 2017), *QYrww.wgp.7A-1, QYrww.wgp.7A-2* (Mu et al., 2020), *QYr.cim-7AS* (Rosewarne et al., 2012), and *QYrst.orr-7AS* (Vazquez et al., 2012), were identified on the short arm of chromosome 7A, and they all conferred APR resistance; therefore, they were different from *QYrpd.swust-7AS*. As a result, *QYrpd.swust-7AS* is likely to be the previously reported QTL *Yr61* based on the origin, effect, resistance type, and linked markers. The relationship between *QYrpd.swust-7AS* derived from Pindong 34 and the previously reported QTL *Yr61* in the region was detected by the two STS markers, which proved the locus was *Yr61*.

#### QYrpd.Swust-7AL

The QTL identified in the present study, *QYrpd.swust-7AL*, confers APR resistance and was located between markers *IWB796* and *IWB14178* (region of 635,495,960 bp−708,137,655 bp) on the long arm of chromosome 7A. Several QTLs have also been previously reported on chromosome 7AL. A locus conferring ASR was controlled by a major gene, *Yrzhong12*-1, located in the interval of SSR markers *Xcfd20* (3.1 cM) and *Xbarc121* (4.9 cM) on chromosome 7AL (Ma et al., 2015). Kanwal et al. (2021) identified the adult-plant stripe rust resistance gene *Yr75* in the Australian wheat cultivar Axe and mapped the gene on the long arm of chromosome 7A, and the two SNP markers *sunKASP_430* and *sunKASP_427* were closely linked to the gene.

#### QYrpd.Swust-7DL

*QYrpd.swust-7DL* was located between markers *IWB13547* and *IWB14996* on the long arm of chromosome 7D and mapped to the 432,435,291 bp−436,968, 553 bp region of the CS genome on chromosome 7DL. To date, only one formally named *Yr* gene, *Yr33*, has been reported on chromosome 7DL (Zahravi et al., 2003). *Yr33* conferred all-stage resistance and was located between markers *Xwgm11* and *Xgwm437*. The *QYr.tam-7D* locus was identified by markers *wPt730042* and *wPt730455*, and it conferred HTAP resistance and was derived from wheat TAM 111 (Basnet et al., 2014a). *QYr.sicau-7D* conferred APR resistance and was located in the QTL region between 4,440,148 bp and 3,937,237 bp (Long et al., 2019). The relationship between *QYrpd.swust-7DL* derived from Pindong 34 and *QYr.sicau-7D* need to be further tested.

### Multi-Environmental Analysis and Digenic Epistatic QTL

Separately, the identified QTLs could not account for the high resistance found in Pindong 34 based on the BIP analysis, which scanned each environment. The MET analysis pointed to additional QTLs that were the same as those detected by the BIP analysis. These QTLs were found due to the high power of multi-environmental analysis, which can detect QTLs (Li et al., 2015). The discovery of 14 marker pairs that show digenic epistatic QTLs (LOD > 15) can complete the picture and explain how Pindong 34 maintains immunity to stripe rust while none of the RILs exhibited this resistance level. The complexity of the genetics underlying Pindong 34 resistance will complicate the transfer of its resistance to other cultivars. Genomic selection methods might help.

## Data Availability Statement

The original contributions presented in the study are included in the article, further inquiries can be directed to the corresponding authors.

## Author Contributions

XZ constructed the RIL population, conducted the experiments, analyzed data, and wrote the manuscript. XL participated in phenotypic and genotypic analyses of the RIL population. DH made the cross and review the manuscript. SY conducted the experiments, analyzed the data, and reviewed the manuscript. ZK conceived the study and reviewed the manuscript. RR analyzed data and wrote the manuscript. All authors approved the final version of the manuscript.

## Funding

This study was financially supported by the Key Research and Development Program of International Science and Technology Innovation Cooperation of Science and Technology Department of Sichuan Province, China (No. 2022YFH0032), and was partially funded by the National Natural Science Foundation of China (No. 32101707), Breakthrough in Wheat Breeding Material and Method Innovation and New Variety Breeding (Breeding Research Project, 2021YFYZ0002), PhD Foundation of Southwest University of Science and Technology (No. 18zx7159, 16zx7162), and Longshan Academic Talent Research Support Program of SWUST (No. 17LZX5).

## Conflict of Interest

The authors declare that the research was conducted in the absence of any commercial or financial relationships that could be construed as a potential conflict of interest.

## Publisher's Note

All claims expressed in this article are solely those of the authors and do not necessarily represent those of their affiliated organizations, or those of the publisher, the editors and the reviewers. Any product that may be evaluated in this article, or claim that may be made by its manufacturer, is not guaranteed or endorsed by the publisher.
